# Supplementary material for: The control of prickle formation in Rubus
Source: bioRxiv. 2026 Mar 12:2025.12.22.695586. Originally published 2025 Dec 23. Preprint. [Version 2] doi: 10.64898/2025.12.22.695586 (PMC12776125; doi:10.64898/2025.12.22.695586)
Supplement: Supplement 7 [file NIHPP2025.12.22.695586v2-supplement-7.pdf]

bioRxiv preprint doi: https://doi.org/10.64898/2025.12.22.695586; this version posted March 12, 2026. The copyright holder for this preprint (which was not certified by peer review) is the author/funder, who has granted bioRxiv a license to display the preprint in perpetuity. It is made available under aCC-BY 4.0 International license.

Supplemental Figure 1 Pedigree of the *s* allele associated with the prickleless phenotype in commercial red raspberry (A) and blackberry (B) accessions used for inheritance-by-descent models.

Supplemental Figure 2 Global genome results for mapping prickleless location A. SNPpoly GWAS results for all chromosomes. B qqPlot

Supplemental Figure 3 Analysis of sequences orthologous to RaWOX1 (Ra_g19519) from *R. arcticus*, *R. lasiococcus*, and *R. pubescens* identified non-synonymous amino acid substitutions within conserved regions of WOX-like proteins. (A) Hydrophilic residue Q>L in smooth *R. arcticus* compared to other *Rubus* WOX1. (B) *R. lasiococcus* has an insertion near the homeodomain in the same position as seen in Riwox1-3 but does not have a stop codon. (C) *R. arcticus*, *R. lasiococcus*, and *R. pubescens* all carry non-synonymous SNPs near the WUS box domain. These substitutions may underlie the smooth phenotype observed in *Cylactis* species

Supplemental Figure 4. Alternate targets for prickleless in blackberry. (A) Phylogenetic tree of orthologs in blackberry to the *AtGLABARA1* gene. The Red box highlights the *GL1* clade. (B) Phylogenetic tree of orthologs in blackberry to the *AtTRANSPARENT TESTA GLABROU*S2 gene. Red box highlights the *AtTTG2* clade. (C) Prickleless phenotype of PW-D0760 plants containing the *35S::GL1-EAR* fusion.

Supplemental Figure 5 Expression of *RhWOX1,* the *Rosa hybrida* ortholog of *RaWOX1,* in the epidermis cells at different stages of prickle development[29]. (A) *RhWOX1* was 20x more highly

bioRxiv preprint doi: https://doi.org/10.64898/2025.12.22.695586; this version posted March 12, 2026. The copyright holder for this preprint (which was not certified by peer review) is the author/funder, who has granted bioRxiv a license to display the preprint in perpetuity. It is made available under aCC-BY 4.0 International license.

Supplemental Tables

Supplemental Table 1

Panel of 268 *Rubus* accessions, including 224 prickled and 44 prickleless accessions from eight subgenera, ensuring a broad representation of genetic and phenotypic variation for the prickle, trichome and glandular trichome traits. Names, plant introduction (PI) number, inventory number, taxon as listed in the USDA-ARS GRIN-GLOBAL database are listed.

Supplemental Table 2

Blackberry accessions in diversity panel with pedigree and predicted zygosity for *s* allele

Supplemental Table 3

Red raspberry accessions in diversity panel with pedigree and predicted zygosity for *s* allele

Supplemental Table 4

Predicted proteins in the *R. ulmifolius* cv. Burbank Thornless genome assembly along with physical positions, closest homolog among the *Rubus argutus* cv. Hillquist genes, and matches obtained from BLASTP analyses with NCBI nr, Araport11, RefSeq, SwissProt and TrEMBL databases as subjects.

Supplemental Table 5

Functional annotations of predicted transcripts in the *R. ulmifolius* cv. Burbank Thornless genome from InterProScan, Gene Ontology (GO), KEGG orthologs, and KEGG pathways.

Supplemental Table 6

Complete list of annotated 62-115 gene models found in mapped 324 Kb region

Supplemental Table 7

Genes downregulated in global expression analysis between prickled and prickleless lines in the PW-D0007 blackberry self-crossed population. Expression analysis revealed 2357 up-regulated genes in the prickled samples and 1921 down-regulated in the prickled samples compared to the prickleless samples.

bioRxiv preprint doi: https://doi.org/10.64898/2025.12.22.695586; this version posted March 12, 2026. The copyright holder for this preprint (which was not certified by peer review) is the author/funder, who has granted bioRxiv a license to display the preprint in perpetuity. It is made available under aCC-BY 4.0 International license.

Supplemental Figure 1

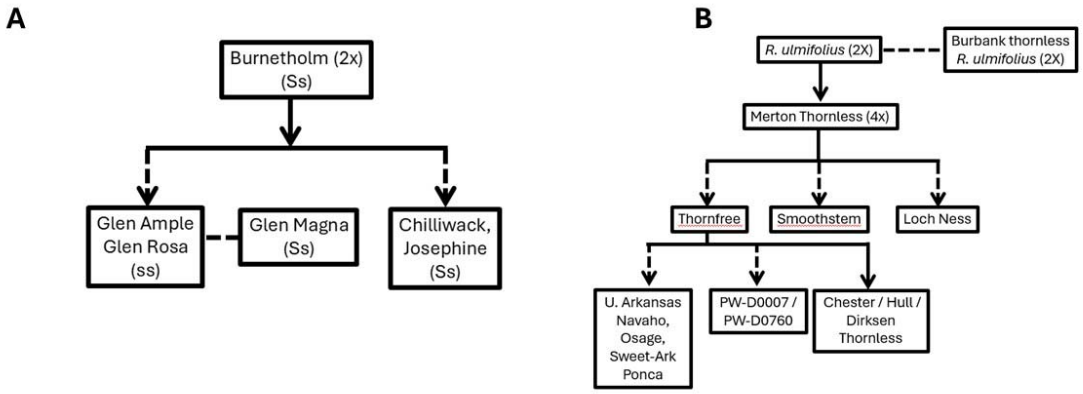

Supplemental Figure 1 Pedigree of the *s* allele associated with the prickleless phenotype in commercial red raspberry (A) and blackberry (B) lines used for inheritance-by-descent models.

bioRxiv preprint doi: https://doi.org/10.64898/2025.12.22.695586; this version posted March 12, 2026. The copyright holder for this preprint (which was not certified by peer review) is the author/funder, who has granted bioRxiv a license to display the preprint in perpetuity. It is made available under aCC-BY 4.0 International license.

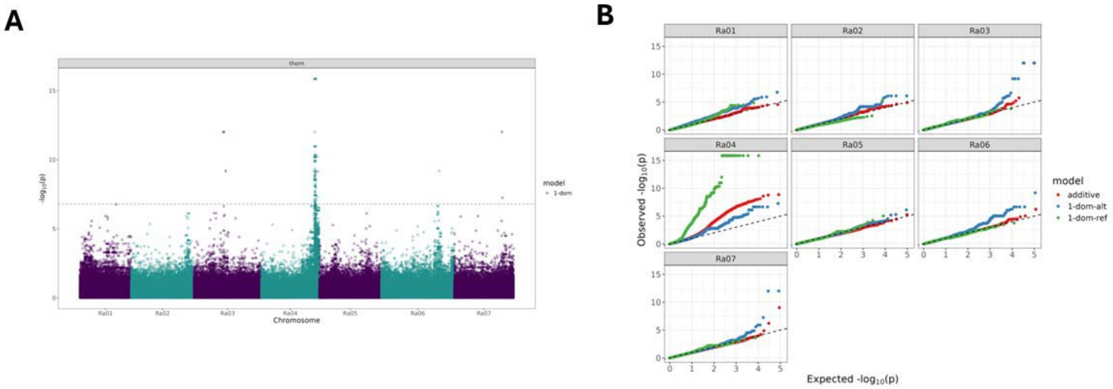

Supplemental Figure 2 A. SNPpoly GWAS results for all chromosomes. B qqPlot

bioRxiv preprint doi: https://doi.org/10.64898/2025.12.22.695586; this version posted March 12, 2026. The copyright holder for this preprint (which was not certified by peer review) is the author/funder, who has granted bioRxiv a license to display the preprint in perpetuity. It is made available under aCC-BY 4.0 International license.

Supplemental Figure 3

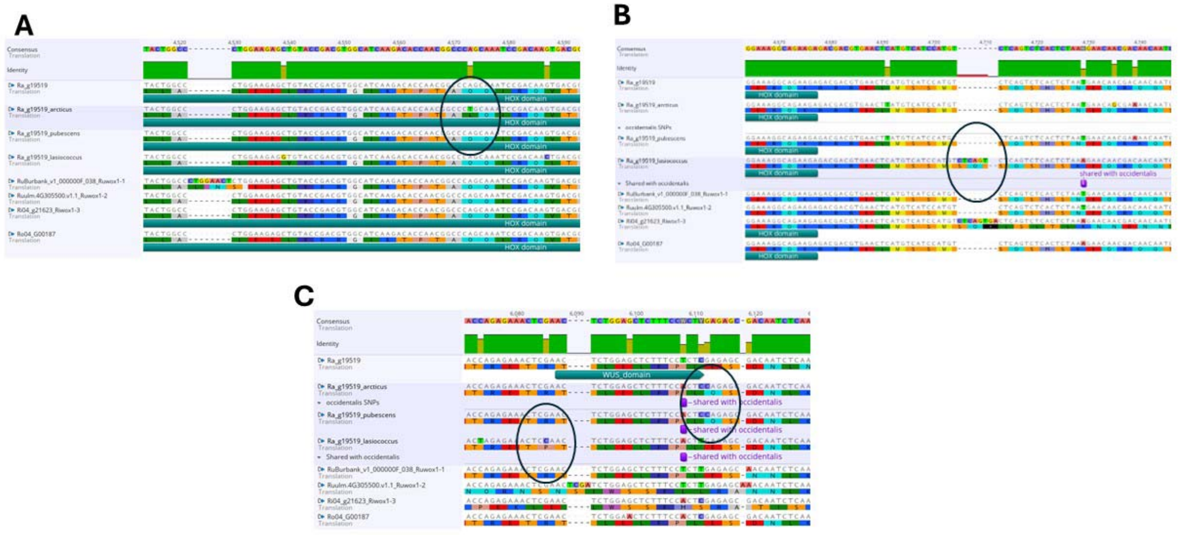

Supplemental Figure 3 Analysis of sequences orthologous *to RaWOX1* (Ra_g19519) from *R. arcticus*, *R. lasiococcus*, and *R. pubescens* identified non-synonymous amino acid substitutions within conserved regions of WOX-like proteins. (A) Hydrophilic residue Q>L in smooth *R. arcticus* compared to other *Rubus* WOX1. (B) *R. Lasiococcus* has insertion near homeodomain in same position as seen in *Riwox1-3* but does not have a stop codon. (C) *R. arcticus, R. lasiococcus, and R. pubescens* all carry non-synonymous SNPs near the WUS box domain. These substitutions may underlie the smooth phenotype observed in these *Cylactis* species

bioRxiv preprint doi: https://doi.org/10.64898/2025.12.22.695586; this version posted March 12, 2026. The copyright holder for this preprint (which was not certified by peer review) is the author/funder, who has granted bioRxiv a license to display the preprint in perpetuity. It is made available under aCC-BY 4.0 International license.

Supplemental Figure 4

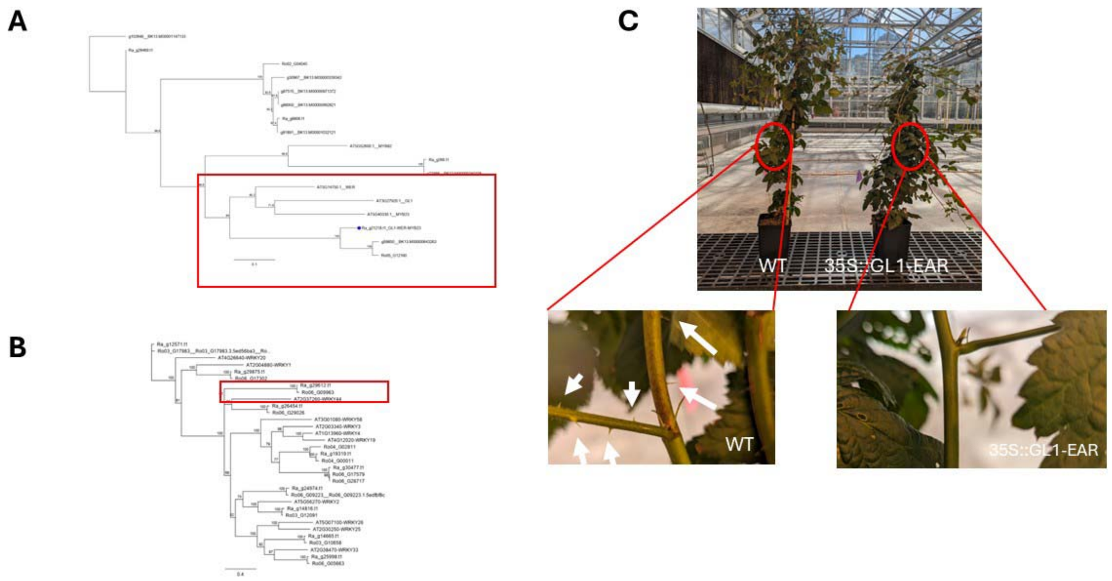

Supplemental Figure 4. Alternate targets for prickleless in Blackberry. (A) Phylogenetic tree of orthologs in blackberry to the *AtGLABARA1* gene. The Red box highlights the *GL1* clade. (B) Phylogenetic tree of orthologs in blackberry to *the AtTRANSPARENT TESTA GLABROUS2* gene. The Red box highlights the *AtTTG2* clade. (C) Prickleless phenotype of PW-D0760 plants containing the *35S::GL1-EAR* fusion.

bioRxiv preprint doi: https://doi.org/10.64898/2025.12.22.695586; this version posted March 12, 2026. The copyright holder for this preprint (which was not certified by peer review) is the author/funder, who has granted bioRxiv a license to display the preprint in perpetuity. It is made available under aCC-BY 4.0 International license.

Supplemental Figure 5

A

Rosa hybrida data	(IP) Initiating prickle	(SP) Soft prickle	(HP) Hard prickle
RhWOX1 expression	low	High (~20x)	low

B

					SP vs IP					
Name	AGI	GeneName	Chromosome	Region	Max group mean	Log_2 fold change	Fold change	*P*-value	FDR *P*-value	Bonferroni
RcHm_v2.0_Chr7g0178511	AT3G18010	WOX1	RcHm_v2.0_Chr7	complement(1164626..1167718)	0.14	4.16	17.82	8.46E-06	7.16E-05	0.43
					HP vs SP					
Name	AGI	GeneName	Chromosome	Region	Max group mean	Log_2 fold change	Fold change	*P*-value	FDR *P*-value	Bonferroni
RcHm_v2.0_Chr7g0178511	AT3G18010	WOX1	RcHm_v2.0_Chr7	complement(1164626..1167718)	0.14	-4.28	-19.49	0.000152	0.000729	1

Supplemental Figure 5 Expression data of *RhWOX1 (RcHm_v2.0_Chr7g0178511),* the *Rosa hybrida* orthologue of *RaWOX1,* in the epidermis cells at different stages of prickle development[29]. (A) *RhWOX1* was 20x more highly expressed in the epidermis of initiating prickles samples compared to initiating epidermis or hard prickle samples. (B) Data taken from Supplemental dataset 1 and 3 comparing differentially expressed genes from each tissue type. Initiating prickle together with bark (IP), soft prickle (SP), hard prickle (HP) - See more details in Figure 1. Swarnkar, M. K., Kumar, P., Dogra, V. & Kumar, S. Prickle morphogenesis in rose is coupled with secondary metabolite accumulation and governed by canonical MBW transcriptional complex. *Plant Direct* **5**, e00325 (2021).

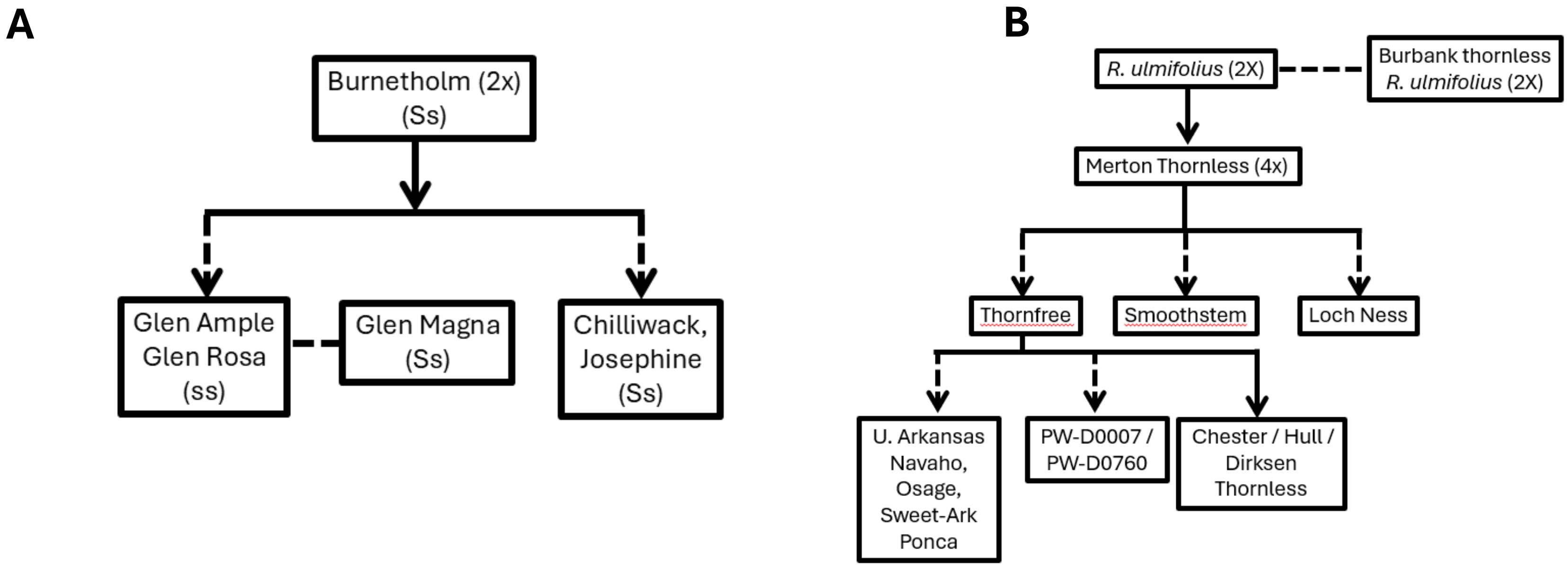
A
Burnetholm (2x)
(Ss)
Glen Ample
Glen Rosa
(ss)
Glen Magna
(Ss)
Chilliwack,
Josephine
(Ss)
B
R. ulmifolius (2X)
Burbank thornless
R. ulmifolius (2X)
Merton Thornless (4x)
Thornfree
Smoothstem
Loch Ness
U. Arkansas
Navaho,
Osage,
Sweet-Ark
Ponca
PW-D0007 /
PW-D0760
Chester / Hull /
Dirksen
Thornless

A

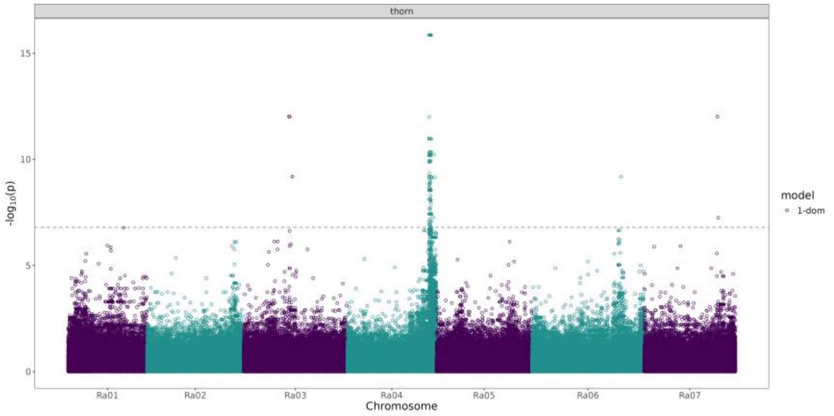
thorn
15
10
5
0
$-\log_{10}(p)$
Ra01
Ra02
Ra03
Ra04
Ra05
Ra06
Ra07
Chromosome
model
1-dom

B

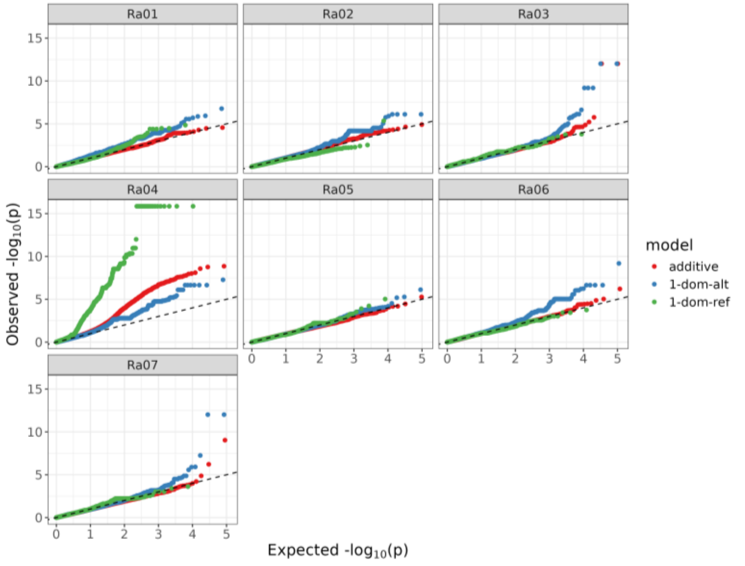
Ra01
Ra02
Ra03
Ra04
Ra05
Ra06
Ra07
Observed $-\log_{10}(p)$
Expected $-\log_{10}(p)$
model
additive
1-dom-alt
1-dom-ref

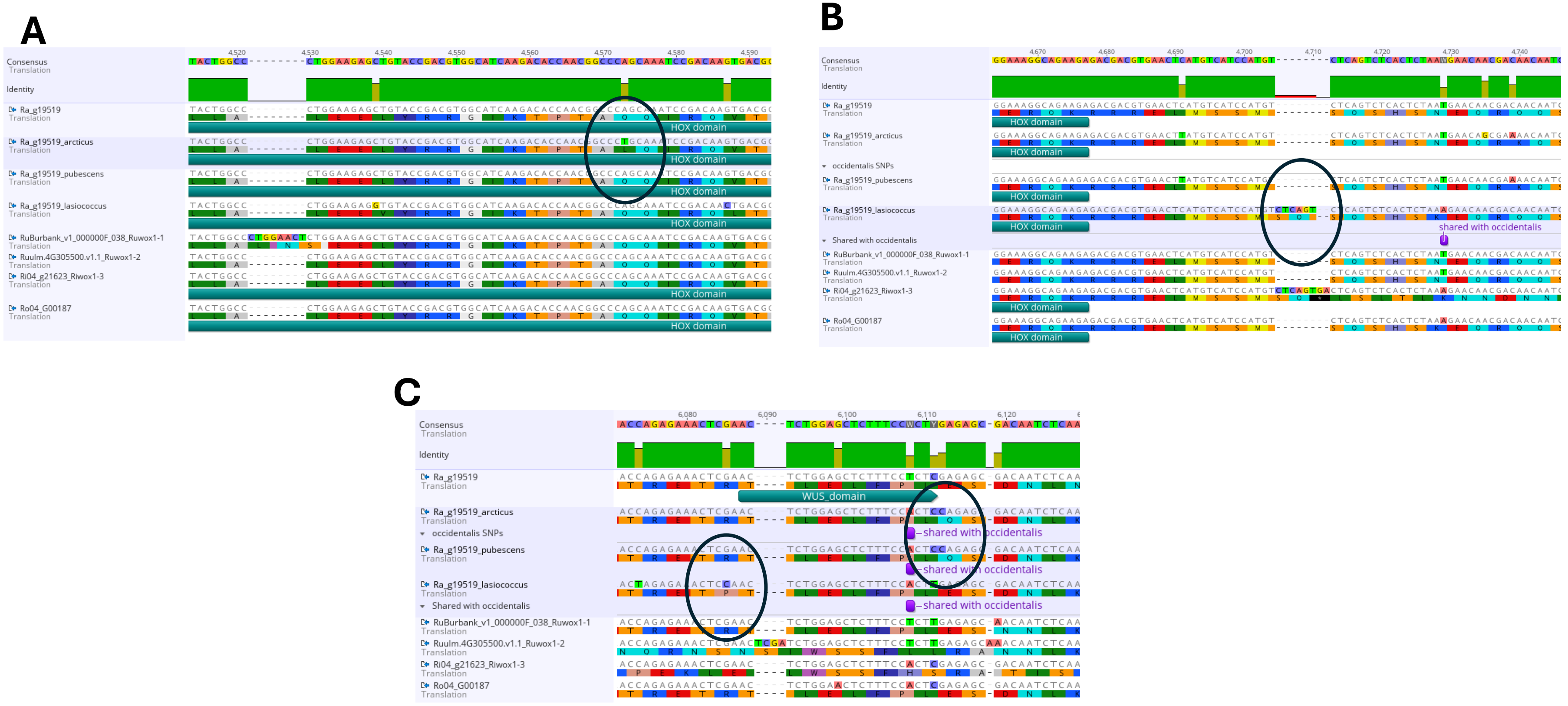
A
B
C
Consensus
Translation
Identity
Ra_g19519
Ra_g19519_arcticus
Ra_g19519_pubescens
Ra_g19519_lasiococcus
RuBurbank_v1_000000F_038_Ruwox1-1
Ruulm.4G305500.v1.1_Ruwox1-2
Ri04_g21623_Riwox1-3
Ro04_G00187
HOX domain
occidentalis SNPs
Shared with occidentalis
shared with occidentalis
WUS_domain

A

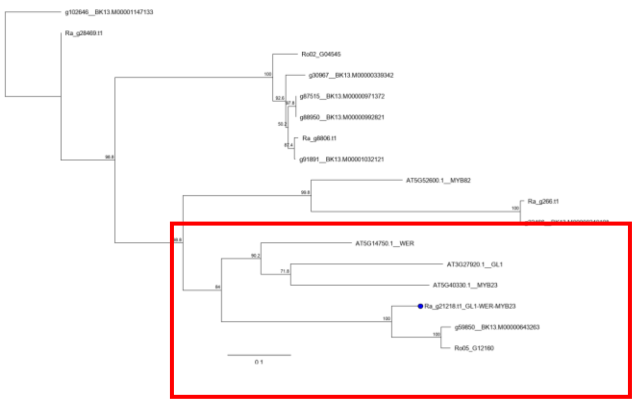
g102646__BK13.M00001147133
Ra_g28469.t1
Ro02_G04545
g30967__BK13.M00000339342
g87515__BK13.M00000971372
g88950__BK13.M00000992821
Ra_g8806.t1
g91891__BK13.M00001032121
AT5G52600.1__MYB82
Ra_g266.t1
AT5G14750.1__WER
AT3G27920.1__GL1
AT5G40330.1__MYB23
Ra_g21218.t1_GL1-WER-MYB23
g59850__BK13.M00000643263
Ro05_G12160
0.1

B

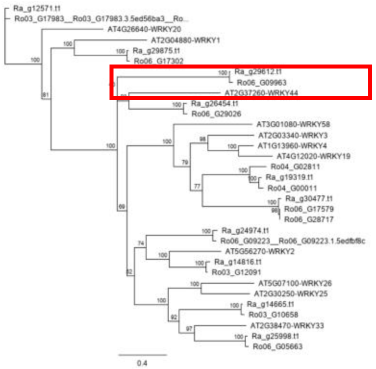
Ra_g12571.t1
Ro03_G17983__Ro03_G17983.3.5ed56ba3__Ro...
AT4G26640-WRKY20
AT2G04880-WRKY1
Ra_g29875.t1
Ro06_G17302
Ra_g29612.t1
Ro06_G09963
AT2G37260-WRKY44
Ra_g26454.t1
Ro06_G29026
AT3G01080-WRKY58
AT2G03340-WRKY3
AT1G13960-WRKY4
AT4G12020-WRKY19
Ro04_G02811
Ra_g19319.t1
Ro04_G00011
Ra_g30477.t1
Ro06_G17579
Ro06_G28717
Ra_g24974.t1
Ro06_G09223__Ro06_G09223.1.5edfbf8c
AT5G56270-WRKY2
Ra_g14816.t1
Ro03_G12091
AT5G07100-WRKY26
AT2G30250-WRKY25
Ra_g14665.t1
Ro03_G10658
AT2G38470-WRKY33
Ra_g25998.t1
Ro06_G05663
0.4

C

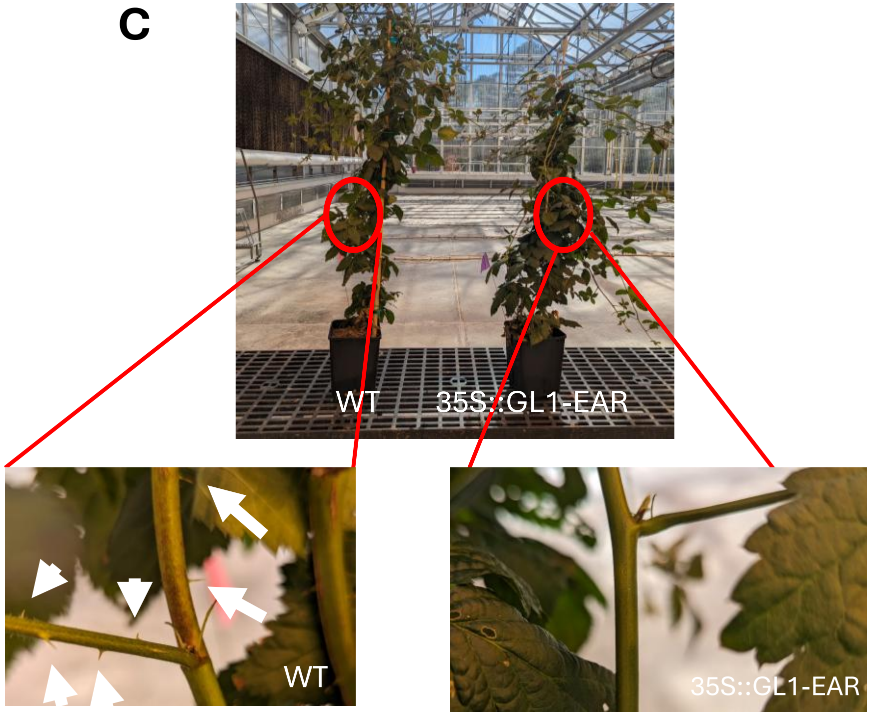
WT
35S::GL1-EAR
WT
35S::GL1-EAR

A

Rosa hybrida data	(IP) Initiating prickle	(SP) Soft prickle	(HP) Hard prickle
RhWOX1 expression	low	High (~20x)	low

B

					SP vs IP					
Name	AGI	GeneName	Chromosome	Region	Max group mean	Log_2 fold change	Fold change	*P*-value	FDR *P*-value	Bonferroni
RcHm_v2.0_Chr7g0178511	AT3G18010	WOX1	RcHm_v2.0_Chr7	complement(1164626..1167718)	0.14	4.16	17.82	8.46E-06	7.16E-05	0.43
					HP vs SP					
Name	AGI	GeneName	Chromosome	Region	Max group mean	Log_2 fold change	Fold change	*P*-value	FDR *P*-value	Bonferroni
RcHm_v2.0_Chr7g0178511	AT3G18010	WOX1	RcHm_v2.0_Chr7	complement(1164626..1167718)	0.14	-4.28	-19.49	0.000152	0.000729	1
