# Supplementary material for: The control of prickle formation in Rubus
Source: bioRxiv. 2025 Dec 23:2025.12.22.695586. Preprint. [Version 1] doi: 10.64898/2025.12.22.695586 (PMC12776125; doi:10.64898/2025.12.22.695586)
Supplement: Supplementary file 1 [file NIHPP2025.12.22.695586v1-supplement-7.pdf]

bioRxiv preprint doi: https://doi.org/10.64898/2025.12.22.695586; this version posted December 23, 2025. The copyright holder for this preprint (which was not certified by peer review) is the author/funder, who has granted bioRxiv a license to display the preprint in perpetuity. It is made available under aCC-BY 4.0 International license.

Supplemental Figure 1

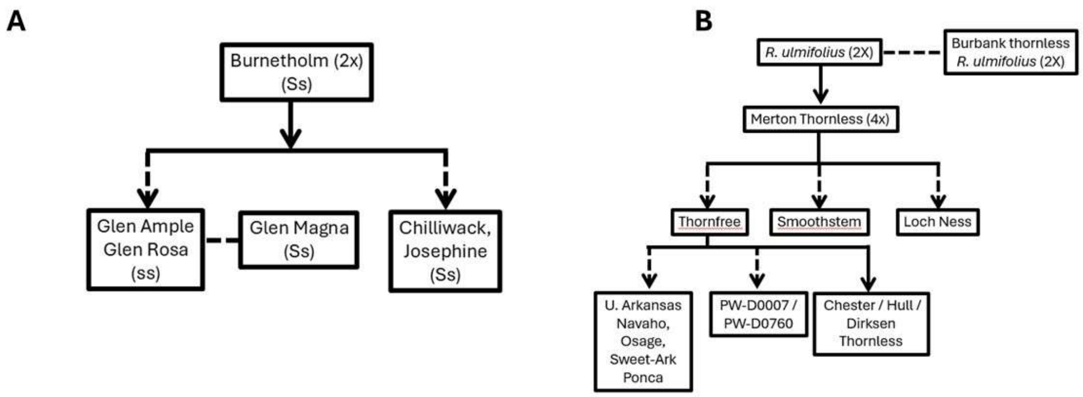

Supplemental Figure 1 Pedigree of the *s* allele associated with the prickleless phenotype in commercial red raspberry (A) and blackberry (B) lines used for inheritance-by-descent models.

bioRxiv preprint doi: https://doi.org/10.64898/2025.12.22.695586; this version posted December 23, 2025. The copyright holder for this preprint (which was not certified by peer review) is the author/funder, who has granted bioRxiv a license to display the preprint in perpetuity. It is made available under aCC-BY 4.0 International license.

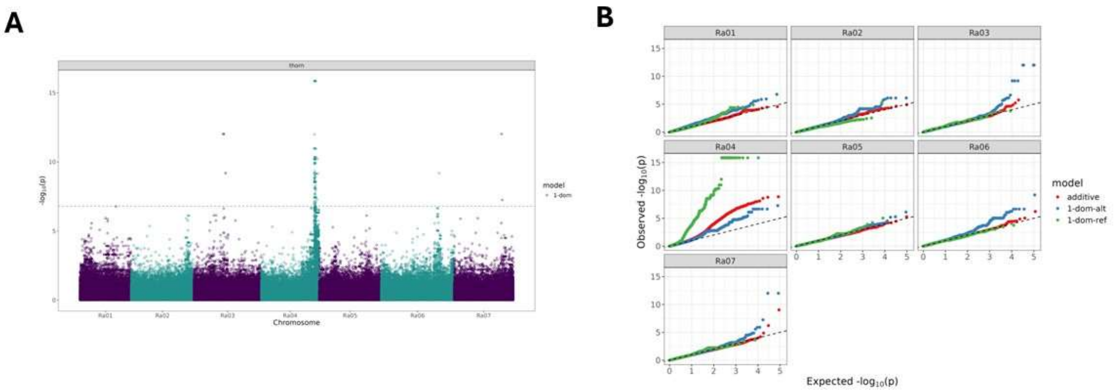

Supplemental Figure 2 A. SNPpoly GWAS results for all chromosomes. B qqPlot

bioRxiv preprint doi: https://doi.org/10.64898/2025.12.22.695586; this version posted December 23, 2025. The copyright holder for this preprint (which was not certified by peer review) is the author/funder, who has granted bioRxiv a license to display the preprint in perpetuity. It is made available under aCC-BY 4.0 International license.

Supplemental Figure 3

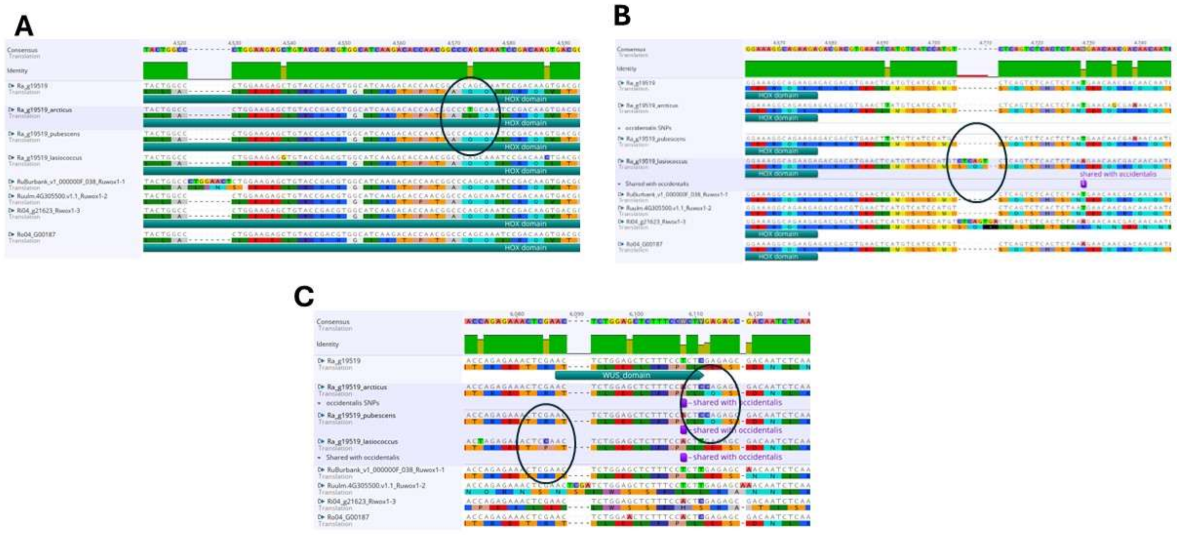

Supplemental Figure 3 Analysis of sequences orthologous *to RaWOX1* (Ra_g19519) from *R. arcticus*, *R. lasiococcus*, and *R. pubescens* identified non-synonymous amino acid substitutions within conserved regions of WOX-like proteins. (A) Hydrophilic residue Q>L in smooth *R. arcticus* compared to other *Rubus* WOX1. (B) *R. Lasiococcus* has insertion near homeodomain in same position as seen in *Riwox1-3* but does not have a stop codon. (C) *R. arcticus, R. lasiococcus, and R. pubescens* all carry non-synonymous SNPs near the WUS box domain. These substitutions may underlie the smooth phenotype observed in these *Cylactis* species

bioRxiv preprint doi: https://doi.org/10.64898/2025.12.22.695586; this version posted December 23, 2025. The copyright holder for this preprint (which was not certified by peer review) is the author/funder, who has granted bioRxiv a license to display the preprint in perpetuity. It is made available under aCC-BY 4.0 International license.

Supplemental Figure 4

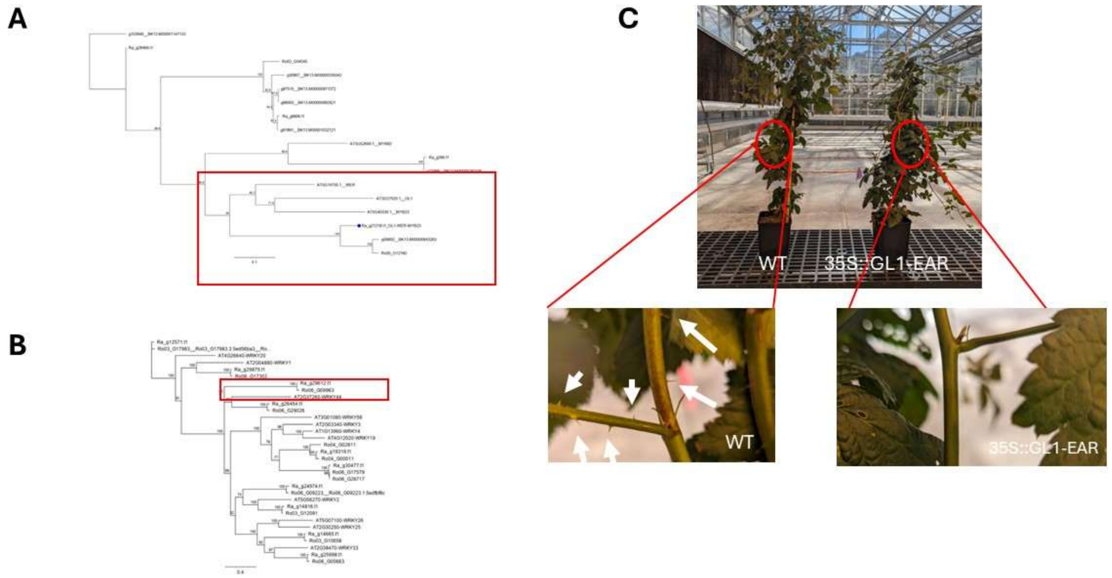

Supplemental Figure 4. Alternate targets for prickleless in Blackberry. (A) Phylogenetic tree of orthologs in blackberry to the *AtGLABARA1* gene. The Red box highlights the *GL1* clade. (B) Phylogenetic tree of orthologs in blackberry to *the AtTRANSPARENT TESTA GLABROUS2* gene. The Red box highlights the *AtTTG2* clade. (C) Prickleless phenotype of PW-D0760 plants containing the *35S::GL1-EAR* fusion.

bioRxiv preprint doi: https://doi.org/10.64898/2025.12.22.695586; this version posted December 23, 2025. The copyright holder for this preprint (which was not certified by peer review) is the author/funder, who has granted bioRxiv a license to display the preprint in perpetuity. It is made available under aCC-BY 4.0 International license.

Supplemental Figure 5

A

Rosa hybrida data	(IP) Initiating prickle	(SP) Soft prickle	(HP) Hard prickle
RhWOX1 expression	low	High (~20x)	low

B

					SP vs IP					
Name	AGI	GeneName	Chromosome	Region	Max group mean	Log_2 fold change	Fold change	*P*-value	FDR *P*-value	Bonferroni
RcHm_v2.0_Chr7g0178511	AT3G18010	WOX1	RcHm_v2.0_Chr7	complement(1164626..1167718)	0.14	4.16	17.82	8.46E-06	7.16E-05	0.43
					HP vs SP					
Name	AGI	GeneName	Chromosome	Region	Max group mean	Log_2 fold change	Fold change	*P*-value	FDR *P*-value	Bonferroni
RcHm_v2.0_Chr7g0178511	AT3G18010	WOX1	RcHm_v2.0_Chr7	complement(1164626..1167718)	0.14	-4.28	-19.49	0.000152	0.000729	1

Supplemental Figure 5 Expression data of *RhWOX1 (RcHm_v2.0_Chr7g0178511),* the *Rosa hybrida* orthologue of *RaWOX1,* in the epidermis cells at different stages of prickle development[29]. (A) *RhWOX1* was 20x more highly expressed in the epidermis of initiating prickles samples compared to initiating epidermis or hard prickle samples. (B) Data taken from Supplemental dataset 1 and 3 comparing differentially expressed genes from each tissue type. Initiating prickle together with bark (IP), soft prickle (SP), hard prickle (HP) - See more details in Figure 1. Swarnkar, M. K., Kumar, P., Dogra, V. & Kumar, S. Prickle morphogenesis in rose is coupled with secondary metabolite accumulation and governed by canonical MBW transcriptional complex. *Plant Direct* **5**, e00325 (2021).
